# The Prognostic Role of Platelet-to-Lymphocyte Ratio in Acute Coronary Syndromes: A Systematic Review and Meta-Analysis

**DOI:** 10.3390/jcm12216903

**Published:** 2023-11-02

**Authors:** Michal Pruc, Frank William Peacock, Zubaid Rafique, Damian Swieczkowski, Krzysztof Kurek, Monika Tomaszewska, Burak Katipoglu, Maciej Koselak, Basar Cander, Lukasz Szarpak

**Affiliations:** 1 Department of Public Health, International European University, 03187 Kyiv, Ukraine; 2 Henry JN Taub Department of Emergency Medicine, Baylor College of Medicine, Houston, TX 77030, USA; 3 Department of Toxicology, Faculty of Pharmacy, Medical University of Gdansk, 80-416 Gdansk, Poland; 4 Department of Clinical Research and Development, LUXMED Group, 02-676 Warsaw, Poland; 5 Department of Emergency Medicine, Ufuk University Medical Faculty, 06510 Ankara, Turkey; 6 Institute of Outcomes Research, Maria Sklodowska-Curie Medical Academy, 00-136 Warsaw, Poland; 7 Department of Emergency Medicine, Bezmialem Vakif University, Fatih, 34093 Istanbul, Turkey

**Keywords:** platelet-to-lymphocyte ratio, PLR, acute coronary syndrome, diagnostic, biomarker, meta-analysis

## Abstract

This study aimed to investigate the potential prognostic role of the platelet-to-lymphocyte (PLR) ratio in patients presenting with suspected acute coronary syndromes (ACS). A systematic search of PubMed Central, Scopus, EMBASE, and the Cochrane Library from conception through 20 August 2023 was conducted. We used odds ratios (OR) as the effect measure with 95% confidence intervals (CIs) for dichotomous data and mean differences (MD) with a 95% CI for continuous data. If I2 was less than 50% or the *p* value of the Q tests was less than 0.05, a random synthesis analysis was conducted. Otherwise, a fixed pooled meta-analysis was performed. Nineteen studies fulfilled the eligibility criteria and were included in the meta-analysis. PLR was higher in MACE-positive (164.0 ± 68.6) than MACE-negative patients (115.3 ± 36.9; MD = 40.14; 95% CI: 22.76 to 57.52; *p* < 0.001). Pooled analysis showed that PLR was higher in AMI patients who died (183.3 ± 30.3), compared to survivors (126.2 ± 16.8; MD = 39.07; 95% CI: 13.30 to 64.84; *p* = 0.003). It was also higher in the ACS vs. control group (168.2 ± 81.1 vs. 131.9 ± 37.7; MD = 39.01; 95% CI: 2.81 to 75.21; *p* = 0.03), STEMI vs. NSTEMI cohort (165.5 ± 92.7 vs. 159.5 ± 87.8; MD = 5.98; 95% CI: −15.09 to 27.04; *p* = 0.58), and MI vs. UAP populations (162.4 ± 90.0 vs. 128.2 ± 64.9; MD = 18.28; 95% CI: −8.16 to 44.71; *p* = 0.18). Overall, our findings confirmed the potential prognostic role of the plate-let-to-lymphocyte (PLR) ratio in patients presenting with suspected acute coronary syndromes (ACS). Its use as a risk stratification tool should be examined prospectively to define its capability for evaluation in cardiovascular patients.

## 1. Introduction

The search for new biomarkers that allow the prediction of disease progression is one of the main areas of development in basic and translational science. One widely discussed biomarker is the platelet-to-lymphocyte ratio (PLR), a simple blood test that measures the relationship between platelets and lymphocytes in the bloodstream. As a marker of inflammation and thus increased predominantly in diseases of pro-inflammatory etiology, the predictive value of PLR shows promise in onco-hematology, immunology, and cardiovascular diseases.

For instance, the utility of PLR as a prognostic biomarker has been evaluated in the area of pulmonary pathology, where Kumar et al. found that an increased PLR was associated with significantly higher 90-day mortality among patients diagnosed with acute exacerbations of chronic obstructive pulmonary disease [1]. However, its prognostic value in pulmonary pathology was confounded by Pertiwi, who reported that in COVID-19 infection, other inflammatory markers, particularly a high CRP, may be more useful in predicting the clinical course [2]. This seeming contradiction of deteriorating prognostic ability of PLR in COVID-19 was addressed by Levy et al., who suggested that the PLR may be more affected by older age and its associated frailty than by the severity of COVID-19 [3].

Consistent with the results of Kumar, a recently published meta-analysis of 32 studies indicated that the PLR obtained at hospital admission correlated with increased mortality. These authors did acknowledge that the studies they included exhibited a high risk of bias and that the overall quality of the evidence was low, such that further investigation was warranted. Finally, a challenging characteristic of newly evaluated biomarkers is the fact that there is also no clear cut-off point for what determines a pathologic PLR, which may explain some variation in reports of patients with a good prognosis and those at a high risk of disease progression [4]. 

PLR is also being studied as a predictor in oncology. Zhang et al. revealed that a high pre-treatment PLR was negatively related to the overall survival and progression-free survival in patients diagnosed with limited-stage small-cell lung cancer [5]. Further, the predictive value of PLR has been investigated in glioblastoma, where a relationship between PLR and overall survival in treatment-naïve patients was reported [6]. Finally, other cancers in which the role and potential utility of PLR are being currently investigated include head and neck squamous cell carcinoma [7], uroepithelial carcinoma [8], gastric cancer [9], and ovarian cancer [10].

Since pro-inflammatory factors have a significant impact on the development of atherosclerotic processes in coronary arteries, PLR is also an object of interest in cardiology. However, despite its pathologic relationship and the fact that it has been widely evaluated, a consensus on its appropriate clinical use is yet to be established. Supporting its application, Pinho et al. found, based on a retrospective analysis, that a high PLR is associated with a higher risk of cardiovascular events in patients with primary hypertension. Further, in heart failure, a high PLR was correlated with the risk of re-hospitalization due to worsening of heart failure, although it was not useful as a diagnostic test [11,12].

Taking into consideration the above, we performed a meta-analysis with the aim of investigating the potential prognostic role of the PLR ratio in patients presenting with suspected acute coronary syndromes (ACS). Compared to previously published papers, new outcomes, e.g., coronary flow vs. no reflow risk, were added to the current meta-analysis.

## 2. Materials and Methods

### 2.1. Search Strategy

We performed this systematic review and meta-analysis based on the Preferred Reporting Items for Systematic Reviews and Meta-Analyses (PRISMA; Table S1) [13]. The protocol was registered with the PROSPERO database (registration number: CRD42023447572).

### 2.2. Literature Enrollment

A literature search was conducted for English-language articles up to 20 August 2023, in the international databases of PubMed Central, Scopus, EMBASE, and the Cochrane Library. The search strategy consisted of variations of the terms “platelet-to-lymphocyte ratio” OR “platelet/lymphocyte ratio” OR “platelet to lymphocyte ratio” OR “platelet lymphocyte ratio” OR “PLR”, AND “acute coronary syndrome” OR “ACS” OR “STEMI” OR “NSTEMI” OR “unstable angina” OR “UA” OR “myocardial infarction” OR “MI”, as both medical subject headings and subject headings specific to each database and keywords or free-text words that included a wide range of derivations to ensure an extensive search was performed. A manual review of the Google Scholar search engine and references to relevant articles was also performed to access any possibly missed studies. It is worth mentioning that the protocol registered in the PROSPERO was not changed during the investigation. 

Year of publication limits were not applied to capture any potential studies that may have been missed in the original review. Searches were limited to English language and adult human subjects. The following inclusion PICOS criteria were applied for the eligible studies: (1) patient, i.e., patients diagnosed with Acute Coronary Syndrome (STEMI, NSTEMI, and unstable angina), (2) intervention, i.e., PLR determination, (3) control, i.e., not applicable, (4) outcomes, i.e., no-flow vs. normal flow after PCI, MACE positive vs. MACE negative, and (5) study, i.e., observational studies (including cross-sectional studies) and non-randomized and randomized clinical trials (if applicable). Meanwhile, the exclusion criteria were as follows: (1) studies in pediatric population/children (age < 18 years); (2) case series, case reports, correspondence, letters to editors, editorials, or review articles; (3) studies that are not available in full-text form or that have not been published; and (4) studies with insufficient data to assess PLR levels.

First, the screening process began by comparing the suitability of the titles and/or abstracts against our eligibility criteria. Any original publications that were cited in the systematic reviews or meta-analyses but missed by the initial search would also be included if they met our inclusion/exclusion criteria. All duplicate articles were removed. The process was then followed by a comprehensive assessment of full-text articles. All of these processes were carried out independently by two reviewers (M.P. and M.T.). If disagreement was found during the screening process, it was resolved by seeking the opinion of a third reviewer (L.S. or D.S.).

### 2.3. Data Extraction and Quality Assessment

Two authors (M.P. and M.T.) separately extracted data from individual reports and recorded the data in an Excel sheet. Any disagreement between the two reviewers in the process of data extraction was resolved by a third reviewer (L.S.). The extracted data included the first author, year of publication, study design, country, sample size, age, gender, comorbidities, ACS types, and PLR values. For publications lacking sufficient information on predictive accuracy to calculate the 2 × 2 contingency tables, we asked the corresponding author for help via email. Studies were excluded if a second email received no response.

Once eligible studies were identified, two researchers (M.P. and D.S.) independently assessed each article for methodological quality and risk of bias using the Newcastle-Ottawa Scale (NOS) [14]. Quality rating disagreements were resolved by discussion with the third researcher (L.S.). The NOS is categorized into three sections: (1) selection of study groups; (2) comparability of groups; and (3) ascertainment of the outcome of interest. The total scores that can be obtained using this tool were in the range of 0–9. A study investigation that obtained a cumulative score of 7 or above was deemed to possess a low likelihood of bias [15]. In cases where a study obtained a cumulative score of 6 or less, it was deemed to possess bias and was excluded from the analysis.

### 2.4. Statistical Analysis

Statistical analysis was conducted with STATA (Software for Statistics and Data Science) software version 17.0 (StataCorp LLC, Lakeway Dr, College Station, TX, USA) and Review Manager software version 5.4 (Nordic Cochrane Centre, Cochrane Collaboration, Copenhagen, Denmark). All statistical tests were two-sided, and the significance level was defined as *p* < 0.05. We used odds ratios (OR) as the effect measure with 95% confidence intervals (CIs) for dichotomous data and mean differences (MD) with a 95% CI for continuous data. If continuous outcomes were reported as median, range, and interquartile range, we estimated means and standard deviations using the formula described by Hozo et al. [16]. The Q test and I^2^ statistics were used to check for heterogeneity. If I^2^ was less than 50% or the *p* value of the Q tests was less than 0.05, a random synthesis analysis was conducted. Otherwise, a fixed pooled meta-analysis was performed [17]. We utilized Egger’s test and funnel plots to check for possible bias and funnel plot tests for asymmetry to assess potential publication bias if more than ten trials were included in a single meta-analysis. A sensitivity analysis using leave-one-out was performed to test for the robustness of the findings.

## 3. Results

### 3.1. Study Selection and Baseline Characteristics

**Figure 1:**
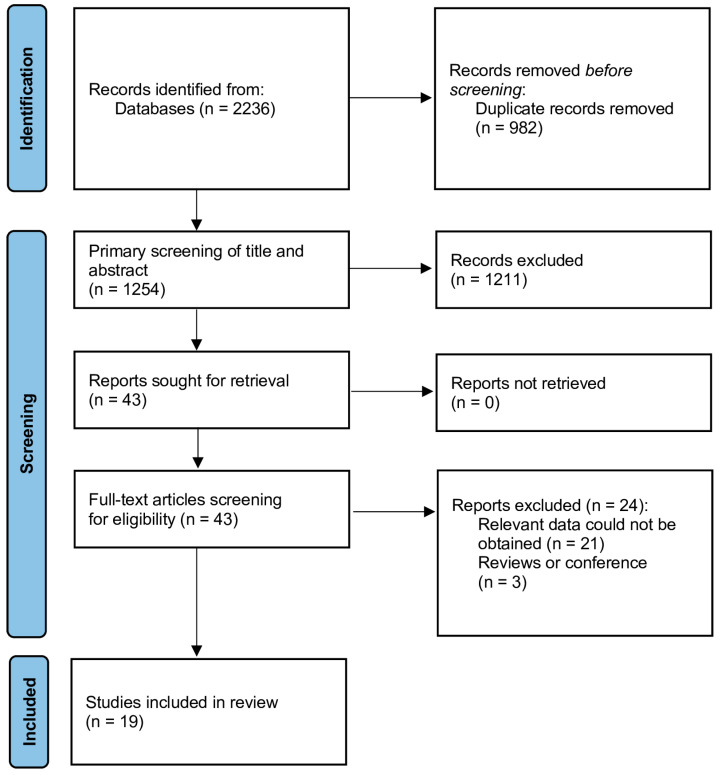
Flow diagram of the search strategy and study selection.

The literature search is illustrated in Figure 1. A total of 2236 records were initially retrieved. Of these, 982 duplicate publications were excluded. After an initial screening of titles and abstracts, 43 articles were selected for assessment of full texts, resulting in a further exclusion of 24 studies. Finally, a total of 19 studies were eventually included in the review (Table 1) [18,19,20,21,22,23,24,25,26,27,28,29,30,31,32,33,34,35,36]. Five prospective and fourteen retrospective studies were published from 2015 to 2023, with sample sizes between 170 and 2230. The participants were from Turkey, Pakistan, China, Poland, Indonesia, Iran, and Yemen. Results of the risk of bias assessment of individual studies can be found in Table 1. All studies were of sufficient quality to be included in the review. Additional information about the studies included in the systematic review is provided in the supplementary file (Table S2). Table S2 includes additional information on the studies: number of patients diagnosed with STEMI, NSTEMI, and UA, detailed comparative characteristics of the study group vs. control group, PLR determination (time), and information on MACE (how MACE was defined in the study).

### 3.2. Meta-Analysis

**Figure 2:**
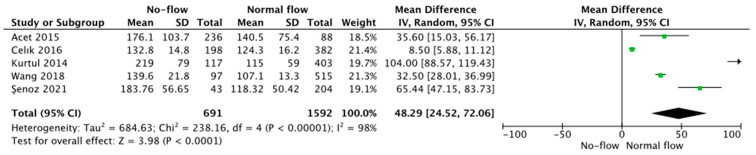
Forest plot of PLR levels among no-flow vs. normal flow acute coronary syndrome patients. The center of each square represents the mean differences for individual trials, and the corresponding horizontal line stands for a 95% confidence interval. The diamonds represent pooled results. Acet et al., 2015 [18]; Celık et al., 2016 [21]; Kurtul et al., 2014 [27]; Wang et al., 2018 [34]; Senoz et al., 2021 [31].

Five trials reported PLR values as related to coronary blood flow and presented the data stratified as no-flow vs. normal flow. Pooled analysis showed that PLR values were higher in those with no vs. normal flow (166.3 ± 77.3 vs. 116.5 ± 43.4, respectively, MD = 48.29; 95% CI: 24.52 to 72.06; *p* < 0.001; Figure 2).

**Figure 3:**
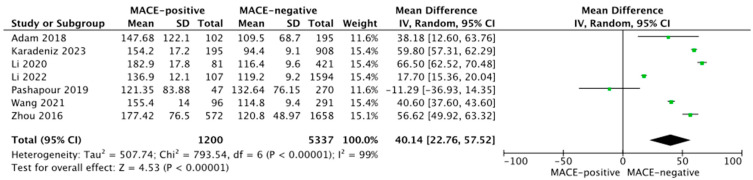
Forest plot of PLR levels among MACE-positive vs. -negative acute coronary syndrome patients. The center of each square represents the mean differences for individual trials, and the corresponding horizontal line stands for a 95% confidence interval. The diamonds represent pooled results. Adam et al., 2018 [19]; Karadeniz et al., 2023 [26]; Li et al., 2020 [28]; Li et al., 2022 [29]; Pashapour et al., 2019 [30]; Wang et al., 2021 [35]; Zhou et al., 2016 [36].

Seven studies reported PLR values in MACE-positive vs. -negative patient groups. Pooled analysis showed PLR was higher in MACE-positive (164.0 ± 68.6) than MACE-negative patients (115.3 ± 36.9; MD = 40.14; 95% CI: 22.76 to 57.52; *p* < 0.001; Figure 3).

Pooled analysis showed that PLR was higher in AMI patients who died (183.3±30.3), compared to survivors (126.2 ± 16.8; MD = 39.07; 95% CI: 13.30 to 64.84; *p* = 0.003). It was also higher in the ACS vs. control group (168.2 ± 81.1 vs. 131.9 ± 37.7; MD = 39.01; 95% CI: 2.81 to 75.21; *p* = 0.03), STEMI vs. NSTEMI cohort (165.5 ± 92.7 vs. 159.5 ± 87.8; MD = 5.98; 95% CI: −15.09 to 27.04; *p* = 0.58), and MI vs. UAP populations (162.4 ± 90.0 vs. 128.2 ± 64.9; MD = 18.28; 95% CI: −8.16 to 44.71; *p* = 0.18). Sensitivity analysis based on the leave-one-out analysis showed that the pooled results were not influenced by a single trial.

## 4. Discussion

Our meta-analysis revealed that the PLR is higher in MACE patients compared to those without MACE. Moreover, the PLR in AMI patients who died was higher than in survivors. In terms of coronary blood flow, the PLR was lower in the normal-flow cohort compared to those with complete blockage. It is worth mentioning that no reflow was found to have a post-PCI TIMI flow grade of 0, 1, or 2, and a TIMI flow grade of 3. 

Some previously conducted studies aimed at detecting the difference between PLR and clinical prognosis in patients with acute coronary syndrome. Li et al. published a meta-analysis demonstrating that individuals with higher PLR had a higher risk of in-hospital adverse outcomes and long-term adverse events [37], including all-cause mortality and CV events among patients with ACS [38]. In a meta-analysis, Dong et al. reported results that were similar. Based on data from 12,619 patients, they found that pre-procedural PLR values were linked to major adverse cardiovascular events in the hospital, death from any cause, and no reflow after percutaneous coronary intervention. Long-term follow-up (up to 82 months after discharge) had similar associations between increased MACE and all-cause mortality [39]. Other meta-analyses [40] have reported findings that are similar.

PLR is not without confounders. Kazem et al. showed that the PLR is an age-independent predictor of cardiovascular mortality, but only in the longer term (adj. HR per 1 SD of 1.04 (95% CI: 1.00–1.08); *p* = 0.039) [41]. PLR may also be important in the prediction of patients with ACS who are more at risk of contrast-induced acute kidney injury (CI-AKI), although, in this case, the prognostic value of the indicator is similar to other biomarkers of inflammation [42,43].

The pathophysiology associated with PLR during atherosclerotic processes in coronary artery disease and acute coronary syndrome is complicated. Reactive platelet activation at the time of ACS may lead to a temporary increase in the absolute platelet count. In turn, the damage resulting from acute myocardial infarction may activate lymphocytes that migrate to the site of injury, consequently reducing their absolute number in the bloodstream. Finally, the stress reaction and systematic inflammation may also disturb the number of morphotic elements. And ultimately, an interaction between lymphocytes and platelets cannot be excluded [44]. Different types of leukocytes contribute to pro-atherosclerotic mechanisms in different ways and to varying degrees. Monocytes, especially in the pro-inflammatory cascade pathway, are the source of Tissue Factor (TF), factor III, which initiates the process of transformation of prothrombin into thrombin. Neutrophils, and to a lesser extent monocytes, release metalloproteinases [45,46,47,48,49]. As a result of apoptosis, e.g., due to sudden hypoxia, neutrophils release neutrophil extracellular traps (NET), an important component of coagulation activation. Endothelial inflammation is indirectly related to the activity of T- and B-lymphocytes. T-lymphocytes activate macrophages, leading to the production of pro-inflammatory cytokines (including IL-1α, IL-6, IL-12, etc.) and tumor necrosis factor (TNF-α), which not only increases inflammation by activating other pro-inflammatory pathways but also hinders healing within the endothelium and contributes to the instability of an atherosclerotic plaque. The role of eosinophils in the development of atherosclerosis is less known. They are suspected of reducing thrombus stability.

In general, the value of the PLR index is related to the immune response, showing the degree of the inflammatory response. An increased PLR index may indicate ongoing pro-inflammatory processes. This, in turn, may contribute to the instability of an atherosclerotic plaque, which may lead to major cardiovascular events. Furthermore, an increased PLR value may result from an increased number of thrombocytes, thereby increasing the risk of thromboembolism. Moreover, an increased number of platelets promotes endothelial damage. A high value of the PLR index may, therefore, be the result of multidirectional changes occurring in the body related to the markers of inflammation [50].

However, before the PLR can be routinely included in the decision-making process, the first challenge is to define a cut-off point for the PLR value that allows the stratification of patients. In addition, further studies need to describe how PLR differs from other similar indicators in practice, e.g., monocyte-to-lymphocyte ratio (MLR), derived neutrophil-to-lymphocyte ratio (dNLR), neutrophil-to-lymphocyte platelet ratio (NLPR), or systemic inflammatory index (SII). Another area of interest may be to define if the systematic immune response can be modified with treatment, if the effect is beneficial, and whether the reduction in inflammation can be measured with the PLR. If so, the PLR biomarker could be used to measure the degree of adherence to therapeutic recommendations or the individual variability of response to the intervention. Single scientific reports show, for example, that ticagrelor may improve the parameters of inflammation compared to another antiplatelet drug, i.e., clopidogrel [51]. Finally, the cost-effectiveness of the PLR has not been defined. If the diagnostic or predictive properties of PLR are confirmed, it may be a cost-effective solution, at least partially limiting the use of expensive technologies, which may be particularly important in rural areas or less developed countries [52].

Other new potential markers include neutrophil-to-lymphocyte ratio, eosinophil-to-leukocyte ratio, eosinophil-to-lymphocyte ratio, eosinophil-to-neutrophil ratio, lymphocyte-to-monocyte ratio, and neutrophil-to-lymphocyte ratio. Patients with acute decompensated heart failure and reduced ejection fraction who experienced a major cardiovascular event (MACE) within 6 months of the index hospitalization had lower eosinophil-to-monocyte ratio and eosinophil-to-lymphocyte ratio values. However, their neutrophil-to-eosinophil ratio and leukocyte-to-eosinophil ratio were higher compared to patients without MACE. Further research is necessary to determine which biomarkers demonstrate the best predictive properties, preferably in a head-to-head comparison [53].

Gender-specific characteristics in the development of the immune system response during the development of cardiovascular diseases remains an area of research. Epidemiological studies have shown that among patients diagnosed with type 2 diabetes, women have a much worse prognosis than men. One hypothesis is that the difference in the immune response between women and men may contribute to a worse prognosis. The increase in the number of platelet–neutrophil conjugates may be one of the factors of the immune response. Also, hormones such as estradiol may determine the specificity of the hormonal response in women. In the context of new biomarkers, careful analyses should be carried out to determine whether there are different cut-offs for women and men [54].

Our meta-analysis has several limitations. These include the lack of a standardized performance platform. This is likely to create variability in comparative numeric values obtained from different data sources. Despite this, we were able to demonstrate clear prognostic associations between abnormal PLR results and both short- and long-term outcomes. Second, we were not able to evaluate the impact of age on outcome events. However, whether age must be included in the interpretation of PLR testing is unclear. Additionally, the effects of hematologic disorders and medications altering hematopoiesis on the PLR accuracy need further evaluation. Furthermore, no study presented here evaluated the consequence of care alterations determined with PLR testing. 

The meta-analysis does not take into account some variables that may affect the usefulness of the PLR biomarker as a predictor. These include diagnosis (STEMI, NSTEMI, UA), how the comparison group was constructed in the studies included in the meta-analysis, the baseline characteristics of the patients, among others. Different ways of describing baseline characteristics, in particular capturing very different information, make it significantly difficult to present the characteristics of participants included in the studies. Moreover, a comparison of morphological and other laboratory parameters between publications would be burdened with a significant systematic error due to the possible different methods of determining parameters and the lack of reference standards in the publications. The meta-analysis did not take into account information about what proportion of patients underwent PCI. Further limitations are associated with the time of PLR determination and MACE definition. To address all the above aspects, Table S2 was added.

Finally, interventional studies, with clinical care changes based on PLR values, are needed before this biomarker can be used in clinical practice.

## 5. Conclusions

This is the largest evaluation of PLR to date. Overall, our findings confirmed the potential prognostic role of the platelet-to-lymphocyte (PLR) ratio in patients presenting with suspected acute coronary syndromes (ACS). Its use as a risk stratification tool should be examined prospectively to define its capability for evaluation in cardiovascular patients. PLR might serve as a surrogate marker of microvascular obstruction and no-reflow risk, with a potential impact on the management of high-risk patients (including gp IIb/IIIa inhibitors). In addition, there is a clinical struggle to identify high-risk patients with no-reflow, which subsequently may contribute to potential efficient strategies to prevent or treat no-reflow, finally leading to short and long-term mortality.

## Figures and Tables

**Table 1:** Baseline characteristics of included trials.

Study	Country	Study Design	Study Group	No of Patient’s	Age, Years	Sex, Male, n, %	NOS Score
Acet et al., 2015 [18]	Turkey	Retrospective study	Normal flow	88	60.2 ± 14.3	72 (81.8%)	9
			No-flow	236	62.1 ± 13.9	165 (69.9%)	
Adam et al., 2018 [19]	Pakistan	Prospective cohort study	MACE-positive	102	54.49 ± 11.41	68 (66.7%)	8
			MACE-negative	195	55.82 ± 10.50	120 (61.5%)	
Cao et al., 2023 [20]	China	Retrospective study	AMI	284	61.27 ± 12.01	237 (83.45%)	9
			No-AMI	91	59.10 ± 11.96	51 (56.04%)	
Celık et al., 2016 [21]	Turkey	Retrospective study	Normal flow	198	62 ± 11	144 (72.7%)	8
			No-flow	382	58 ± 12	307 (80.4%)	
Chen et al., 2023 [22]	China	Single-center, observational, retro- spective study	AMI With Death	94	76.61 ± 11.98	53 (56.38%)	8
			AMI Without Death	94	76.88 ± 9.04	48 (51.06%)	
Dziedzic et al., 2023 [23]	Poland	Retrospective study	AMI	103	73.1 ± 9.9	NS	8
			No-AMI	135	71.1 ± 8.3	NS	
Guclu et al., 2020 [24]	Turkey	Prospective cohort study	AMI With Death	22	68.09 + 18.7	10 (45.5%)	9
			AMI Without Death	148	61.5 + 10.6	120 (81.1%)	
Harun et al., 2016 [25]	Indonesia	Retrospective study	AMI	223	NS	151 (67.71%)	7
			No-AMI	198	NS	84 (42.42%)	
Karadeniz et al., 2023 [26]	Turkey	Retrospective, cross-sectional study	MACE-positive	195	65.3 ± 10.8	123 (63.1%)	8
			MACE-negative	908	76.6 ± 11.3	636 (70.0%)	
Kurtul et al., 2014 [27]	Turkey	Retrospective, cross-sectional study	Normal flow	403	58 ± 12	311 (77.2%)	8
			No-flow	117	68 ± 13	74 (63.2%)	
Li et al., 2020 [28]	China	Retrospective study	MACE-positive	81	NS	NS	7
			MACE-negative	421	NS	NS	
Li et al., 2022 [29]	China	Single-center prospective observational study	MACE-positive	107	NS	78 (72.9%)	8
			MACE-negative	1594	NS	1227 (77.0%)	
Pashapour et al., 2019 [30]	Iran	Retrospective, cross-sectional study	MACE-positive	47	63.13 ± 13.14	38 (80.9%)	8
			MACE-negative	270	59.54 ± 11.70	225 (83.3%)	
Senoz et al., 2021 [31]	Turkey	Retrospective study	Normal flow	204	57.29 ± 13.14	152 (74.5%)	9
			No-flow	43	61.74 ± 12.47	27 (62.8%)	
Sheng et al., 2021 [32]	China	Prospective cohort study	STEMI	24	71.1 ± 9.8	12 (50.0%)	8
			NSTEMI	25	66.5 ± 10.8	18 (72.0%)	
			UA	156	63.6 ± 10.6	108 (69.5%)	
Shumilah et al., 2021 [33]	Yemen	Case–control study	AMI	100	55.5 ± 15	60 (60.0%)	7
			No-AMI	100	54.1 ± 15	60 (60.0%)	
Wang et al., 2018 [34]	China	Retrospective study	Normal flow	515	62 ± 13	368 (71.5%)	8
			No-flow	97	63 ± 16	29 (69.1%)	
Wang et al., 2021 [35]	China	Prospective observational study	MACE-positive	96	71 (63~78)	NS	7
			MACE-negative	291	67 (59~75)	NS	
Zhou et al., 2016 [36]	China	Retrospective study	MACE-positive	572	62.93 ± 11.18	361 (63.11%)	8
			MACE-negative	1658	58.12 ± 11.31	934 (56.33%)	

Legend: AMI: acute myocardial infarction; DM: diabetes mellitus; MACE: major advance cardiac event; NOS: Newcastle Ottawa Scale; NS: not specified; and UA: unstable angina.

## Data Availability

The data that support the findings of this study are available on request from the corresponding author (L.S.).

## Supplementary Materials

The following supporting information can be downloaded at: https://www.mdpi.com/article/10.3390/jcm12216903/s1, Table S1: PRISMA checklist; Table S2. Detailed characteristics of the studies included in the meta-analysis.
