# Supplementary material for: The Prognostic Role of Platelet-to-Lymphocyte Ratio in Acute Coronary Syndromes: A Systematic Review and Meta-Analysis
Source: J Clin Med. 2023 Nov 2;12(21):6903. doi: 10.3390/jcm12216903 (PMC10650024; doi:10.3390/jcm12216903)
Supplement: Supplementary file 1 [file jcm-12-06903-s001.zip › jcm-2680154-supplementary.pdf]

The prognostic role of platelet-to-lymphocyte ratio in acute coronary syndromes: a systematic review and meta-analysis

Supplementary Digital File

Table S1. PRISMA checklist

Section and Topic	Item #	Checklist item	Location where item is reported
**TITLE**			
Title	1	Identify the report as a systematic review.	1
**ABSTRACT**			
Abstract	2	See the PRISMA 2020 for Abstracts checklist.	1
**INTRODUCTION**			
Rationale	3	Describe the rationale for the review in the context of existing knowledge.	1,2
Objectives	4	Provide an explicit statement of the objective(s) or question(s) the review addresses.	2
**METHODS**			
Eligibility criteria	5	Specify the inclusion and exclusion criteria for the review and how studies were grouped for the syntheses.	2
Information sources	6	Specify all databases, registers, websites, organisations, reference lists and other sources searched or consulted to identify studies. Specify the date when each source was last searched or consulted.	2
Search strategy	7	Present the full search strategies for all databases, registers and websites, including any filters and limits used.	2,3
Selection process	8	Specify the methods used to decide whether a study met the inclusion criteria of the review, including how many reviewers screened each record and each report retrieved, whether they worked independently, and if applicable, details of automation tools used in the process.	3
Data collection process	9	Specify the methods used to collect data from reports, including how many reviewers collected data from each report, whether they worked independently, any processes for obtaining or confirming data from study investigators, and if applicable, details of automation tools used in the process.	3
Data items	10a	List and define all outcomes for which data were sought. Specify whether all results that were compatible with each outcome domain in each study were sought (e.g. for all measures, time points, analyses), and if not, the methods used to decide which results to collect.	3
	10b	List and define all other variables for which data were sought (e.g. participant and intervention characteristics, funding sources). Describe any assumptions made about any missing or unclear information.	3
Study risk of bias assessment	11	Specify the methods used to assess risk of bias in the included studies, including details of the tool(s) used, how many reviewers assessed each study and whether they worked independently, and if applicable, details of automation tools used in the process.	3
Effect measures	12	Specify for each outcome the effect measure(s) (e.g. risk ratio, mean difference) used in the synthesis or presentation of results.	3
Synthesis methods	13a	Describe the processes used to decide which studies were eligible for each synthesis (e.g. tabulating the study intervention characteristics and comparing against the planned groups for each synthesis (item #5)).	3
	13b	Describe any methods required to prepare the data for presentation or synthesis, such as handling of missing summary statistics, or data conversions.	3
	13c	Describe any methods used to tabulate or visually display results of individual studies and syntheses.	3
	13d	Describe any methods used to synthesize results and provide a rationale for the choice(s). If meta-analysis was performed, describe the	3

Section and Topic	Item #	Checklist item	Location where item is reported
		model(s), method(s) to identify the presence and extent of statistical heterogeneity, and software package(s) used.	
	13e	Describe any methods used to explore possible causes of heterogeneity among study results (e.g. subgroup analysis, meta-regression).	3
	13f	Describe any sensitivity analyses conducted to assess robustness of the synthesized results.	3
Reporting bias assessment	14	Describe any methods used to assess risk of bias due to missing results in a synthesis (arising from reporting biases).	3
Certainty assessment	15	Describe any methods used to assess certainty (or confidence) in the body of evidence for an outcome.	3
**RESULTS**			
Study selection	16a	Describe the results of the search and selection process, from the number of records identified in the search to the number of studies included in the review, ideally using a flow diagram.	4
	16b	Cite studies that might appear to meet the inclusion criteria, but which were excluded, and explain why they were excluded.	4
Study characteristics	17	Cite each included study and present its characteristics.	4-6
Risk of bias in studies	18	Present assessments of risk of bias for each included study.	5,6
Results of individual studies	19	For all outcomes, present, for each study: (a) summary statistics for each group (where appropriate) and (b) an effect estimate and its precision (e.g. confidence/credible interval), ideally using structured tables or plots.	4,6,7
Results of syntheses	20a	For each synthesis, briefly summarise the characteristics and risk of bias among contributing studies.	7
	20b	Present results of all statistical syntheses conducted. If meta-analysis was done, present for each the summary estimate and its precision (e.g. confidence/credible interval) and measures of statistical heterogeneity. If comparing groups, describe the direction of the effect.	4,6,7
	20c	Present results of all investigations of possible causes of heterogeneity among study results.	4,6,7
	20d	Present results of all sensitivity analyses conducted to assess the robustness of the synthesized results.	7
Reporting biases	21	Present assessments of risk of bias due to missing results (arising from reporting biases) for each synthesis assessed.	7
Certainty of evidence	22	Present assessments of certainty (or confidence) in the body of evidence for each outcome assessed.	7
**DISCUSSION**			
Discussion	23a	Provide a general interpretation of the results in the context of other evidence.	7
	23b	Discuss any limitations of the evidence included in the review.	8
	23c	Discuss any limitations of the review processes used.	8
	23d	Discuss implications of the results for practice, policy, and future research.	8
**OTHER INFORMATION**			

Section and Topic	Item #	Checklist item	Location where item is reported
Registration and protocol	24a	Provide registration information for the review, including register name and registration number, or state that the review was not registered.	2
	24b	Indicate where the review protocol can be accessed, or state that a protocol was not prepared.	2
	24c	Describe and explain any amendments to information provided at registration or in the protocol.	2
Support	25	Describe sources of financial or non-financial support for the review, and the role of the funders or sponsors in the review.	8
Competing interests	26	Declare any competing interests of review authors.	8
Availability of data, code and other materials	27	Report which of the following are publicly available and where they can be found: template data collection forms; data extracted from included studies; data used for all analyses; analytic code; any other materials used in the review.	8

Table S2. Detailed characteristics of the studies included in the meta-analysis.

	Study	STEMI	NSTEMI	UA	Study vs control group	PLR determination	MACE determination	MACE examples
1.	Acet et al., 2015	324	0	0	SR - spontaneous reperfusion<br><br>Vs. non-SR - non spontaneous reperfusion	Before receiving primary PCI	In hospital follow-up period	cardiogenic shock, new advanced heart failure, pulmonary edema, complete atrioventricular block (AVB) requiring a temporary pacemaker, severe ventricular arrhythmia, major bleeding requiring blood transfusion, and in-hospital mortality during the post-PCI follow-up period
2.	Adam et al., 2018	118	79	100	white blood cell (WMR)<br><br>WMR ≤ 1000)<br><br>Vs. WMR > 1000)	At baseline, within 30 minutes from hospital admission	30-days follow-up	non-fatal MI, re-hospitalization, cardiac arrhythmias and death
3.	Cao et al., 2023	No information	No information	No information	AMI vs. Non-AMI	After an overnight fasting	Not applicable	Not applicable
4.	Celık et al., 2016	580	0	0	No reflow (a post-PCI TIMI flow grade of 0, 1 or 2) vs. reflow (TIMI flow grade 3)	Immediately after obtaining ECG	In-hospital	in-stent thrombosis, nonfatal MI, and in-hospital mortality during the in-hospital follow-u period
5.	Chen et al., 2023	898	2348	0	With death vs. without death	After hospital admission (routine procedure)	In-hospital	Not applicable
6.	Dziedzic et al., 2023	No information	No information	No information	ACS vs. stable CAD	At hospital admission	Not applicable	Not applicable
7.	Guclu et al., 2020	0	170	0	Non-mortality vs. mortality	After hospital admission (routine procedure)	1 year follow-up	Death
8.	Harun et al., 2016	66	68	89	ACS vs. healthy controls	After hospital admission (routine procedure)	Not applicable	Not applicable

9.	Karadeniz et al., 2023	403	700	0	MACE vs. non-MACE (long-term)	Within the first hour of admission	In hospital and 50-months follow-up	Long-term MACE were defined as mortality, re-infarction and target vessel revascularization.
10.	Kurtul et al., 2014	520	0	0	Normal-reflow group (post-intervention TIMI 3) vs. none-reflow group (post-intervention TIMI flow grade of 0, 1 or 2)	Admission in the emergency room	Not applicable	Not applicable
11.	Li et al., 2020	No information	No information	No information	MACE vs. non-MACE	Morning of the second day after admission	In-hospital	Acute cardiac failure, severe arrhythmias (ventricular tachycardia/ventricular fibrillation and severe conduction block), non-fatal myocardial infarction, and death
12.	Li et al., 2022	218	216	1267	Not applicable	After 12 hours of fasting, after hospital admission	Medical follow-up of 30 months	All-cause death, non-fatal ischemic stroke, non-fatal MI
13.	Pashapour et al., 2019	317	0	0	ST segment resolution: i) STR of lower than 50% (STR < 50%), ii) STR in the range of 50%-70% (51% < STR < 70%), iii) STR of higher than 70% (STR > 70%)	After hospital admission, no specific information regarding time	In-hospital	MACE has generally been defined as all-cause heart failure, mortality as the result of cardiac diseases, and reinfarction
14.	Senoz et al., 2021	247	0	0	No-reflow (TIMI 0, 1, 2) vs. normal flow (TIMI 3)	After hospital admission in emergency room or coronary care unit before coronary angiography	Not applicable	Not applicable
15.	Sheng et al., 2021	24	25	156	STEMI vs. NSTEMI vs. UA	After hospital admission and before PCI, at 24	On average 15 months after PCI	cardiovascular death, new myocardial infarction, unplanned PCI, and

						hours and 30 days after PCI		progression to class IV heart failure according to NYHA
16.	Shumilah et al., 2021	No information	No information	No information	100 patients with ACS (STEMI, NSTEMI, UA) vs. 100 healthy controls	At hospital admission	Not applicable	Not applicable
17.	Wang et al., 2018	612	0	0	No-reflow (TIMI 0, 1, 2) vs. normal reflow (TIMI 3)	After admission in emergency room	Not applicable	Not applicable
18.	Wang et al., 2021	387	0	0	MACE vs. non-MACE	After hospital admission	6 months after discharge	Heart failure, non-fatal re-infarction, recurrent angina pain, re-hospitalization for cardiovascular-related illness, repeat percutaneous coronary intervention (PCI), coronary artery bypass grafting, and all-cause mortality
19.	Zhou et al., 2016	No information	No information	No information	MACE vs. non-MACE	At hospital admission	At 1, 3, 6, 12, 24, 36, 48, 60 and 72 months (median duration of 58 months) after discharge	CVD events, including nonfatal MI, cardiovascular death, unstable angina, nonfatal ischemic stroke and revascularization procedure, are a composite of clinical events and end points of ACS
